# Electronic Structure and Carrier Mobilities of Arsenene and Antimonene Nanoribbons: A First-Principle Study

**DOI:** 10.1186/s11671-015-0955-7

**Published:** 2015-06-04

**Authors:** Yanli Wang, Yi Ding

**Affiliations:** Department of Physics, Center for Optoelectronics Materials and Devices, Zhejiang Sci-Tech University, Hangzhou, 310018 People’s Republic of China; Department of Physics, Hangzhou Normal University, Hangzhou, 310036 People’s Republic of China

**Keywords:** As/Sb nanostrcuture, Tunable gap variation, Deformation potential theory

## Abstract

Arsenene and antimonene, i.e. two-dimensional (2D) As and Sb monolayers, are the recently proposed cousins of phosphorene (Angew. Chem. Int. Ed., 54, 3112 (2015)). Through first-principle calculations, we systematically investigate electronic and transport properties of the corresponding As and Sb nanoribbons, which are cut from the arsenene and antimonene nanosheets. We find that different from the 2D systems, band features of As and Sb nanoribbons are dependent on edge shapes. All armchair As/Sb nanoribbons keep the indirect band gap feature, while the zigzag ones transfer to direct semiconductors. Quantum confinement in nanoribbons enhances the gap sizes, for which both the armchair and zigzag ones have a gap scaling rule inversely proportional to the ribbon width. Comparing to phosphorene, the large deformation potential constants in the As and Sb nanoribbons cause small carrier mobilities in the orders of magnitude of 10^1^–10^2^ cm^2^/Vs. Our study demonstrates that the nanostructures of group-Vb elements would possess different electronic properties for the P, As, and Sb ones, which have diverse potential applications for nanoelectronics and nanodevices.

## Background

Since the discovery of phosphorene, two-dimensional (2D) group-V nanostructures have attracted lots of interests from physicists, chemists, and material scientists [1–5]. The 2D phosphorene nanosheet, i.e. the black-P monolayer, is a direct semiconductor with a high hole mobility comparable to graphene [6]. When cut into nanoribbons, both the zigzag and armchair black-P nanoribbons (zPNRs, aPNRs) keep the direct band gap feature, which maintain high carrier mobilities akin to the 2D nanosheet [7–12]. The quantum confinement effects in nanoribbons further enhance the band gaps of PNRs, whose gap sizes are monotonously decreased with the increasing ribbon width [13–16].

Very recently, arsenene and antimonene, which are single-atom-thick layers of arsenic and antimony, have been proposed as new members of group-V nanostructures [17–21]. These As/Sb nanosheets have a different buckling structure from phosphorene, which prefer to the blue-P-like structure rather than the black-P one [18, 19]. It results in an indirect band gap in arsenene and antimonene nanosheets [17]. For the corresponding arsenene and antimonene nanoribbons (AsNRs, SbNRs), their electronic and transport properties are still unknown so far. Do these AsNRs and SbNRs have different band features from the nanosheets? Can they have high hole mobilities akin to phosphorene? To address these questions, we preform a first-principle investigation on the electronic structures and carrier mobilities of arsenene and antimonene nanoribbons.

## Methods

The first-principle calculations are performed by the VASP code within the Perdew-Burke-Ernzerhof (PBE) projector augmented wave pseudopotentials and plane-wave basis sets of 400 eV cutoff energy [22]. A *k*-mesh of 1×5×1 / 1×7×1 is utilized in the relaxation for the armchair/zigzag nanoribbons, and the *k*-mesh grid is increased to 1×15×1 and 1×25×1 in the static and band calculations, respectively. A vacuum layer of more than 12.5 Åis used to simulate the isolated nanoribbons. The lattice constants along the periodic directions and all the internal coordinates are optimized until the convergence of the force on each atom is less than 0.01 eV/Å. The hybrid exchange and correlation functional of Heyd-Scuseria-Ernzerhof (HSE) has been used to check the obtained results. In the calculations, the HSE06 form with a screening parameter of 0.11 bohr^−1^ is used, and the HSE calculations are done by the FHI-aims code [23].

## Results and Discussion

Firstly, to verify the accuracy of our calculations, we perform investigations on the arsenene and antimonene nanosheets. Our calculated results show that both arsenene and antimonene have the chair-buckled honeycombs, in which the buckling height between two sublattices are up to 1.40 and 1.64 Å, respectively. The buckled structures suggest that different from graphene, arsenene and antimonene prefer the sp^3^ hybridization. The in-plane lattice constants are 3.60 and 4.12 Åfor arsenene and antimonene. Indirect semiconducting behaviours are found in these nanosheets, for which the valence band maximum (VBM) is at the *Γ* point and the conduction band minimum (CBM) is along the *Γ*−*M* line. The calculated PBE band gaps are 1.59 and 1.28 eV for As and Sb nanosheets, respectively. All these structural and electronic properties of arsenene and antimonene agree well with previous studies [17–19].

**Fig. 1:**
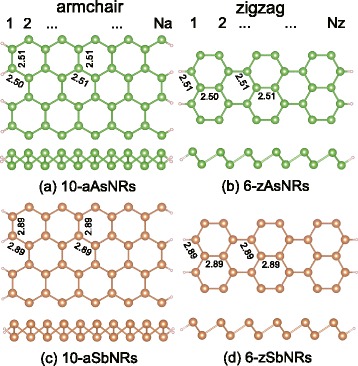
The top and lateral view of armchair and zigzag **a**, **b** As and **c**, **d** Sb nanoribbons. The *N*_*a*_ (*N*_*z*_) corresponds to the number of the dimer lines (*zigzag chains*) across the nanoribbon width. The bond lengths in the centre/edges of nanoribbons are indicated

Since the chair-buckled structures in arsenene and antimonene are analogous to blue-P and silicene ones [24–26], following previous convention of nanoribbons [27], the armchair (a) and zigzag (z) As/Sb nanoribbons are constructed by cutting the sheets along the $<1\overline {1}00>$ and $<2\overline {1}\overline {1}0>$ directions. As shown in Fig. 1, the edge atoms are passivated by H atoms, which eliminate the dangling bonds and strengthen the stabilities of edges [13].

**Fig. 2:**
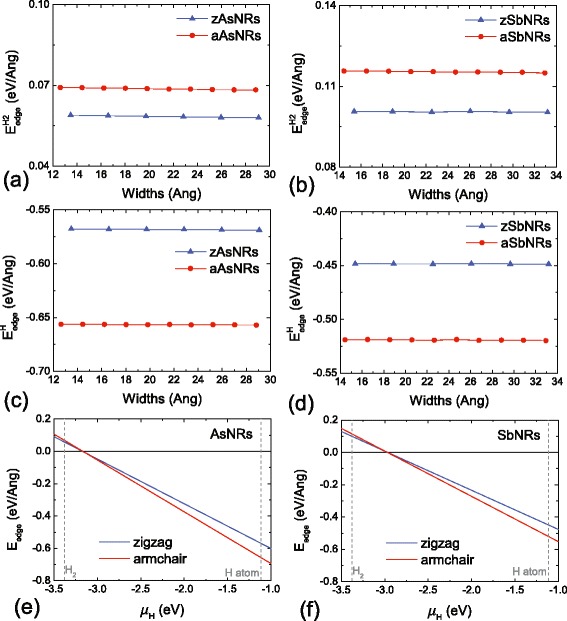
The edge energies of AsNRs under the **a** hydrogen-poor and **c** hydrogen-rich conditions. The edge energies of SbNRs under the **b** hydrogen-poor and **d** hydrogen-rich condition. The functions of edge energies versus the chemical potentials are depicted in **e** and **f** for the AsNRs and SbNRs, respectively

**Fig. 3:**
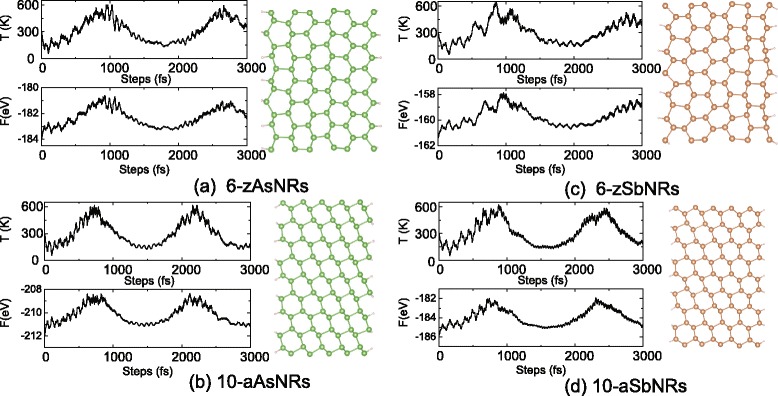
The fluctuations of temperature and free energy, and the final structures of the AIMD simulations for the **a** 6-zAsNRs, **b** 10-aAsNRs, **c** 6-zSbNRs, and **d** 10-aSbNRs

In order to determine the stabilities of these AsNRs and SbNRs, the edge energies (*E*_edge_) of different nanoribbons are calculated as *E*_edge_=(*E*_*X*NRs_−*n*_*X*_*μ*_*X*_−*n*_*H*_*μ*_*H*_)/2*L*_edge_, where *X* = As or Sb, and *E*_*X*NRs_ is the total energy of corresponding As and Sb nanoribbons. *n*_*X*_ is the number of As or Sb atoms in the nanoribbons, and the *μ*_*X*_ is the chemical potential for it, which is adopted to the energy of 2D nanosheets. *n*_*H*_ is the number of H atoms in the edge. The *L*_edge_ is the length of edges, and the factor of 2 is from the two edges of nanoribbons. Normally, the edge energies are dependent on the chemical potential of hydrogen, and the values of *μ*_*H*_ can be varied for the different surroundings of nanoribbons. Here, we have considered the typical hydrogen-poor and hydrogen-rich cases in Fig. 2a–d. The general variations of edge energies versus the chemical potential are depicted in Fig. 2e, f. Under the hydrogen-poor condition, the *μ*_*H*_ is adopted to the energy of a H _2_ molecule, the obtained results as $E_{\text {edge}}^{H2}$ are depicted in Fig. 2a, b. While for the hydrogen-rich surrounding, we use the atomic energy of an isolate H atom for the *μ*_*H*_, and the corresponding results of $E_{\text {edge}}^{H}$ are shown in Fig. 2c, d. It can be seen that all the $E_{\text {edge}}^{H2}$ are positive, suggesting the hydrogen-poor surrounding is unfavourable for the AsNRs and SbNRs. While for the $E_{\text {edge}}^{H}$, they become negative to −0.5∼−0.6 eV/AA, which indicates that the hydrogen-rich condition would benefit the formation of As and Sb nanoribbons. Besides that, we have also performed the ab initio molecular dynamics (AIMD) simulations on the AsNRs and SbNRs to check their thermodynamic stabilities of AsNRs and SbNRs. The AIMD simulations are carried out at the temperature of 300 K by the Nose thermostat with a step time of 1 fs. As shown in Fig. 3, after 3000 time steps, both the AsNRs and SbNRs are just distorted slightly. The hydrogen passivations are still preserved, and no edge reconstructions occur. The final configurations of AIMD simulations can go back to the initial optimized structures after a full structural relaxation. Therefore, combining the calculated edge energies, it can be concluded that both As and Sb nanoribbons have robust stabilities under the hydrogen-rich condition and can maintain the hydrogen-passivated structures at room temperature.

**Fig. 4:**
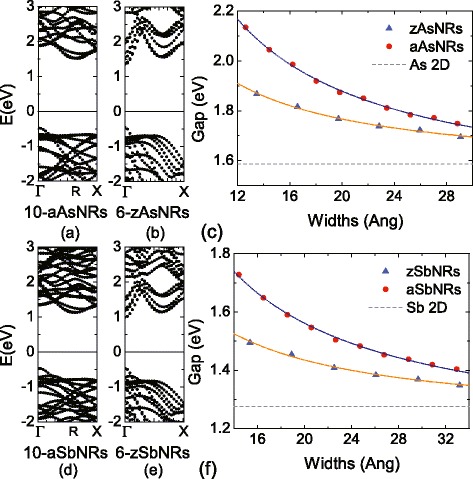
The band structures of **a** 10-aAsNRs, **b** 6-zAsNRs, **d** 10-aSbNRs, and **e** 6-zSbNRs, in which the Fermi level is adopted to 0 eV. The variations of band gaps as a function of ribbon widths for **c** As and **f** Sb nanoribbons. The *dot lines* correspond to the fittings of data

**Fig. 5:**
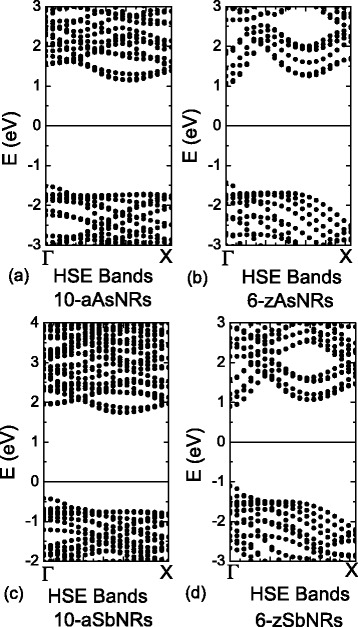
The HSE band structures of **a**, **b** As and **c**, **d** Sb nanoribbons

Figure 4 depicts the typical band structures of AsNRs and SbNRs, which clearly show the similarity between the As and Sb cases. Thus, we take the 10-aAsNRs and 6-zAsNRs as an example. All of them are nonmagnetic confirmed by a further spin-polarized calculation. The 10-aAsNRs are indirect band gap semiconductors, whose VBM is at the *Γ* point, while the CBM is at the *R* point (about 0.6*π*/*a*) along *Γ*−*X* line. The band gap is 1.99 eV for 10-aAsNRs, which is a bit larger than the 2D value of arsenene. While for 6-zAsNRs, they become direct band gap semiconductors with a PBE gap of 1.82 eV. The VBM and CBM are both at the *Γ* point as shown in Fig. 4b. We also check these results by a hybrid calculation with HSE functional. As shown in Fig. 5, the HSE calculations have found the band gaps are increased to 2.66 and 2.48 eV for 10-aAsNRs and 6-zAsNRs, but the band features are still indirect and direct for armchair and zigzag ones, respectively. Thus, although the band gaps are underestimated by PBE calculations, the basic physics and predicted properties would be same for the PBE and HSE functionals.

**Fig. 6:**
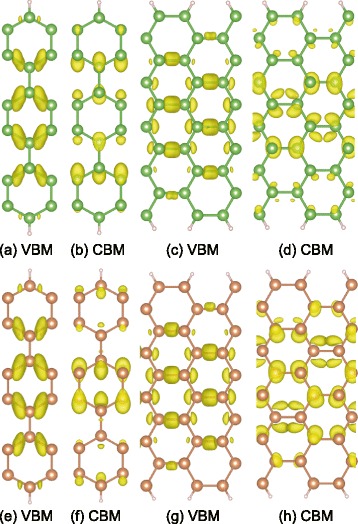
The distributions of partial charge densities for VBM and CBM in **a**, **b** 6-zAsNRs, **c**, **d** 10-aAsNRs, **e**, **f** 6-zSbNRs, and **g**, **h** 10-aSbNRs, in which the isosurfaces are adopted to 30 % of their maximums

Figure 6 depicts the partial charge densities of VBM and CBM for the AsNRs and SbNRs. It shows that the VBM and CBM are mainly distributed in the middle of nanoribbons, while the edge atoms are less involved. For the 6-zAsNRs, the VBM and CBM correspond to bonding and anti-bonding states along the zigzag chains. Similarly, in the 10-aAsNRs, they are also the bonding and anti-bonding states along the dimer lines as shown in Fig. 6c, d. A further partial charge density analysis shows the bottom conduction band at the *Γ* point is a nearly free electron (NFE)-like state for 10-aAsNRs. This NFE-like state has a higher energy than the anti-bonding state, causing the indirect band gap in 10-aAsNRs as shown in Fig. 4a. In our calculations, we find that all the aAsNRs and aSbNRs are indirect semiconductors, while the zAsNRs and zSbNRs are direct ones. This suggests that once cutting into nanoribbons, the edge shapes can be used to switch the band features of arsenene and antimonene. This will be an advantage over the phosphorene nanoribbons, which always keep the same band feature as its 2D nanosheets. The black-P nanoribbons are all direct semiconductors as the black-P monolayer [13], while the blue-P nanoribbons are all indirect ones like the blue-P sheet [28]. Here, for the As and Sb nanoribbons, they can possess different band features from arsenene and antimonene sheets.

Besides that, since the spacial distribution of VBM and CBM is limited by the ribbon width, quantum confinement effect is appreciable on the gaps of AsNRs and SbNRs. Figure 4c, f plots the gap variations of AsNRs and SbNRs versus the ribbon width. It shows that the gap sizes are gradually decreased with the increasing ribbon widths, and the varying trend can be fit as *Δ*_*G*_=*Δ*_0_+*γ*/*W* [28]. Here, *Δ*_*G*_ is the band gap, *Δ*_0_ is a fit parameter, which is expected to close to the 2D value of nanosheets, *W* is the ribbon width, and *γ* is a parameter representing the strength of quantum confinement. For AsNRs, we obtain the *Δ*_0_= 1.44 (1.55) eV and *γ*= 8.67 (4.29) eV/Åfor armchair (zigzag) ones. Similarly, the aSbNRs (zSbNRs) have the *Δ*_0_= 1.15 (1.22) eV and *γ*= 8.28 (4.23) eV/Å. These obtained *γ* values indicate that the quantum confinement is stronger in the armchair nanoribbons than in the zigzag ones for both arsenene and antimonene, which results in larger gaps in the armchair case. The *Δ*_0_ of aAsNRs and aSbNRs deviate from their 2D value by about 0.14 and 0.13 eV, indicating the edge states still slightly affect the band gaps. While for the zAsNRs and zSbNRs, their *Δ*_0_ converges to the 2D values within −0.03 and −0.05 eV, suggesting the weak effect of zigzag edges.

Then, we investigate the hole mobilities of AsNRs and SbNRs. Following the deformation potential theory [29], the mobility *μ*_1*D*_ in one-dimensional systems can be evaluated as

$$\mu_{1D}=\sqrt{\frac{2}{\pi k_{B}T}}\frac{e \hbar^{2} C_{1D}}{m^{*3/2}E^{2}}. $$

**Fig. 7:**
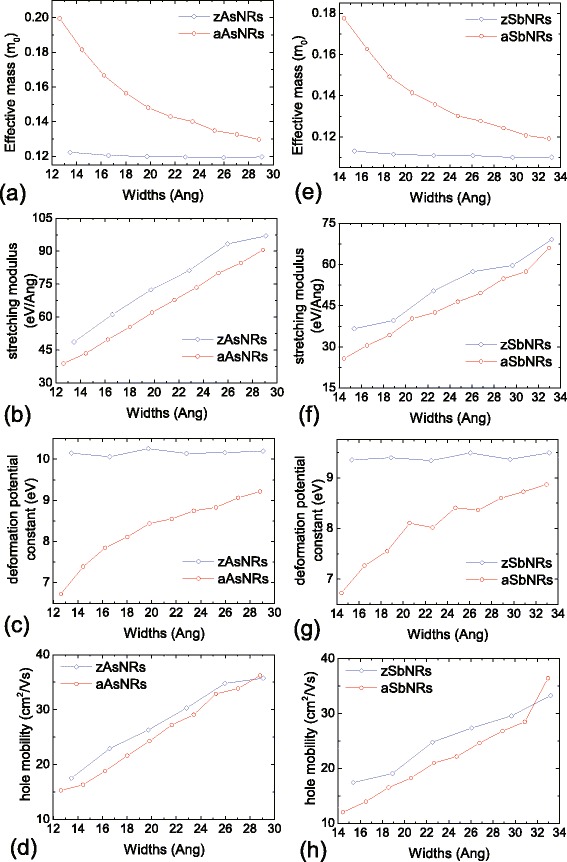
The effective mass of hole, the stretching modulus, the deformation potential constant of VBM, and hole mobility for **a**–**d** As and **e**–**h** Sb nanoribbons. *m*_0_ in **a** and **e** is the mass of free electron

**Fig. 8:**
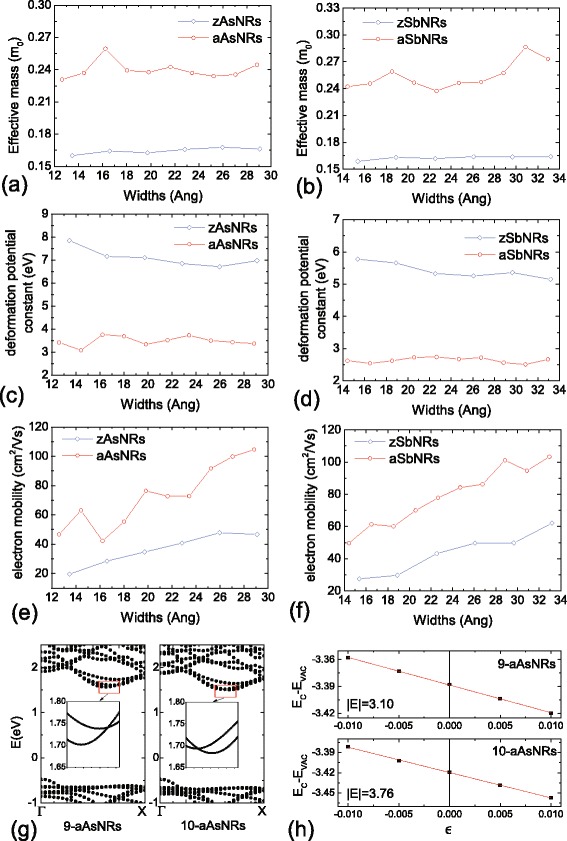
**a**–**f** Effective masses of electron, deformation potential constants of CBM, and electron mobilities for As and Sb nanoribbons. **g** The PBE band structures of 9-aAsNRs and 10-aAsNRs, in which the band edges around the CBM are zoomed in. **h** The fitting process of deformation potential constants for 9-aAsNRs and 10-aAsNRs

Here, *C*_1*D*_ is the stretching modulus, *m*^∗^ is the effective mass of hole, and *E* is the deformation potential constant of VBM for holes. It should be noted that in this formula, only the longitudinal acoustic phonon scattering is included in the calculation of charge mobility. Thus, the obtained *μ*_1*D*_ are intrinsic charge mobilities, which have been generally used as valid evaluations for nanostructures [6]. Figure 7a–d shows the results of AsNRs. The *m*^∗^ is small in the zAsNRs, which is about 0.12 *m*_0_ regardless of the ribbon width. While for the aAsNRs, the *m*^∗^ is decreased from 0.20 *m*_0_ in 8-aAsNR to 0.13 *m*_0_ in 17-aAsNR. The *C*_1*D*_ of zAsNRs and aAsNRs are monotonously increased with the ribbon width as shown in Fig. 7b. However, the *E* is large in AsNRs, which is about 10 eV for the zigzag case and 6.7–9.2 eV for the armchair ones. Such large *E* is attributed to the spacial distribution of VBM in AsNRs. As indicated in Fig. 6a, c, they are located at the As-As bonds along the ribbon direction, which are sensitive to the axial strain. In phosphorene, a small *E* of 0.15 eV results a high hole mobility up to 10^4^ cm^2^/Vs [6], while the large *E* in AsNRs reduces the hole mobilities down to 15–36 cm^2^/Vs. Similar phenomenon also occurs in SbNRs, whose hole mobilities are 12–36 cm^2^/Vs as shown in Fig. 7e–h. Besides that, we also study the electron mobilities of AsNRs and SbNRs in the work. As shown in Fig. 8, effective masses of electrons are similar between the As and Sb nanoribbons, which are about 0.24 *m*_0_ and 0.16 *m*_0_ for the armchair and zigzag ones, respectively. The corresponding deformation potential constants of CBM are 7–8 eV and 3–4 eV in zAsNRs and aAsNRs, respectively, and they become smaller in the Sb ones, which are *E* = 5–6 and 2–3 eV in zSbNRs and aSbNRs, respectively. The corresponding electron mobilities are calculated as 19–47 cm^2^/Vs in zAsNRs, and they are increased to 42–104 cm^2^/Vs in aAsNRs. Similarly, the electron mobilities are 27–62 and 50–103 cm^2^/Vs in zSbNRs and aSbNRs, respectively. It should be noted that comparing to the hole mobilities of nanoribbons in Fig. 7, there are more fluctuations in the electron mobilities, especially for the aAsNR case. This is due to that at the bottom of conduction bands, some of them are nearly degenerate in these nanoribbons. As shown in Fig. 8g, h, although these bands have similar band dispersions, the effective masses and deformation potential constants become a bit different. For example, in 9-aAsNRs, the *m*^∗^ and *E* of electrons are 0.236 *m*_0_ and 3.10 eV, respectively, while in 10-aAsNRs, they become 0.259 *m*_0_ and 3.76 eV, respectively. Since the mobility values are sensitive to these data, i.e. *μ*_1*D*_∝*m*^∗−3/2^*E*^−2^, the obtained electron mobilities are varied fluctuantly for these armchair nanoribbons.

Finally, utilizing the deformation potential theory [6], the 2D carrier mobilities of As and Sb nanosheets are calculated as

$$\mu_{2D}=\frac{e \hbar^{3} C_{2D}}{k_{B}Tm^{*}\overline{m^{*}}E^{2}}. $$

**Fig. 9:**
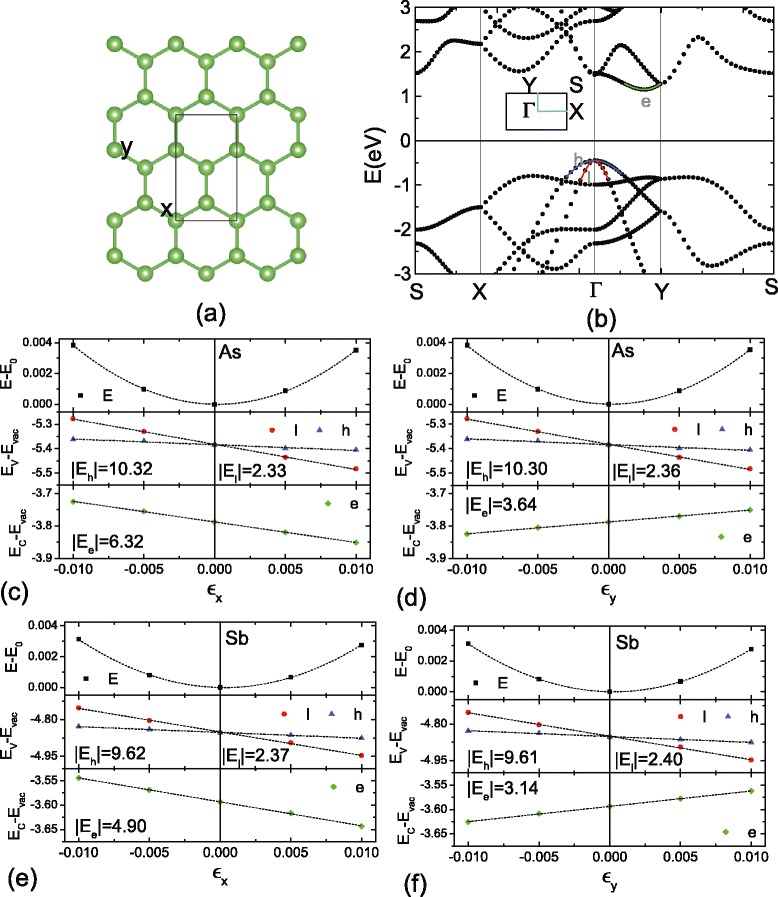
**a** The used rectangular supercell for 2D mobility calculations, in which the *x* and *y* directions corresponds to the zigzag and armchair orientations of sheet, respectively. **b** The corresponding PBE band structure of As sheet, where band edges for the heavy/light hole (*h*/*l*) and electron (*e*) are marked. **c**, **d** The variations of total energies, absolute energies of VBM and CBM of As nanosheet versus *x* and *y* directional strains. **e**, **f** Similar to **c**, **d**, but the data is adopted to the Sb case. In **c**–**f**, the absolute values of deformation potential constant, i.e. the fitted slope, are also marked

Comparing to the 1D formula, here, the *C*_2*D*_ becomes the elastic modulus of sheet, which is obtained from the relationship of (*E*_*ε*_−*E*_0_)/*S*_0_=1/2*C*_2*D*_*ε*^2^. $\overline {m^{*}}$ is the average effective mass of carrier, which is adopted to the value of $\sqrt {m_{x}m_{y}}$ [6]. In the calculations, as shown in Fig. 9a, a rectangular $1\times \sqrt {3}$ supercell is used for the As and Sb nanosheets. Figure 9b depicts the corresponding PBE band structures of As sheet from the supercell calculations. The VBM is still located at the *Γ* point, which is doubly degenerate similar to the results from the primitive cell calculations [17]. While due to the band folding of supercell, the CBM moves into the line between the *Γ* and *Y* points. Effective masses are nearly isotropic for the holes in As sheets, which are *m*_*hx*_/ *m*_*hy*_= 0.48/0.50 *m*_0_ for the heavy one and *m*_*lx*_/ *m*_*ly*_= 0.10/0.09 *m*_0_ for the light one. While for the electron, effective masses become anisotropic as *m*_*ex*_= 0.17 and *m*_*ey*_= 0.50 *m*_0_. Through the fitting processes in Fig. 9c, d, the *C*_2*D*_ is obtained as 52 J/m^2^ for both *x* and *y* directions. The |*E*| for the holes are also isotropic in the As sheet, which are about 2.3 and 10.3 eV for the heavy and light holes, respectively. While for the electrons, the anisotropic |*E*| are 6.3 and 3.6 eV along the *x* and *y* directions. Using the above data, the *μ*_2*D*_ are obtained as ∼0.88/0.81 and 1.10/1.22 ×10^3^ cm^2^/Vs for heavy and light holes along the *x*/*y* direction, and the 2D electron mobilities are 0.57 and 0.58 ×10^3^ cm^2^/Vs for the *x* and *y* directions, respectively. For the Sb nanosheet, by similar approach shown in Fig. 9e, f, we obtain *μ*_2*D*_ are 0.59/0.55 and 0.79/0.87 ×10^3^ cm^2^/Vs for heavy and light holes, and 0.57/0.54 ×10^3^ cm^2^/Vs for the electron along the *x*/*y* direction. Comparing to phosphorene, which has a high carrier mobility up to 10^4^ cm^2^/Vs, the mobilities of As and Sb sheets are much smaller. This is attributed to the large deformation potential constant *E* in the As and Sb sheets. Based on the formula of mobilities, it can be seen that the *μ* is inverse to *E*^2^. In the As and Sb sheets, the *E* is about 3–10 eV, which is one order of magnitude larger than that of the phosphorene monolayer (0.15 eV [6]). In addition, the As and Sb nanosheets are also softer than the P one, which results in smaller *C*_2*D*_ values (52/32 J/m^2^ for As/Sb ones) than the P (101.60 J/m^2^ for phosphorene [6]). Thus, although the effective masses are small in the As and Sb sheets, their carrier mobilities will be lower than the phosphorene one.

## Conclusions

In summary, we investigate the electronic properties of arsenene and antimonene nanoribbons. It is found that for both arsenene and antimonene, the armchair nanoribbons are indirect semiconductors, while the zigzag nanoribbons are direct ones regardless of the ribbon width. The quantum confinement is stronger in the armchair nanoribbons than in the zigzag ones, causing bigger band gaps in the armchair case. Due to the special charge distributions of band edges, As and Sb nanoribbons have large deformation potential constants, which result in a conventional carrier mobility in the magnitude of 10^1^–10^2^ cm^2^/Vs. Similarly, owing to the same reason, the carrier mobilities of 2D arsenene and antimonene nanosheets are only 0.5–1.2 ×10^3^ cm^2^/Vs, which are much lower than the phosphorene value. Due to the shape-dependent band features and large deformation potential constants, the arsenene- and antimonene-based nanomaterials will have potential applications in nanoelectronics and nanodevices.
